# Clinical recommendations to diagnose and monitor patients with transthyretin amyloid cardiomyopathy in Asia

**DOI:** 10.1002/clc.23882

**Published:** 2022-07-06

**Authors:** Weiqin Lin, Pairoj Chattranukulchai, Alex PW Lee, Yen‐Hung Lin, Wen‐Chung Yu, Houng‐Bang Liew, Abraham Oomman

**Affiliations:** ^1^ Department of Cardiology National University Heart Centre Singapore; ^2^ Yong Loo Lin School of Medicine National University of Singapore Singapore; ^3^ Division of Cardiovascular Medicine, Department of Medicine, Faculty of Medicine, King Chulalongkorn Memorial Hospital Chulalongkorn University Bangkok Thailand; ^4^ Division of Cardiology, Department of Medicine and Therapeutics, Li Ka Shing Institutes of Health Science, Prince of Wales Hospital The Chinese University of Hong Kong Hong Kong China; ^5^ Division of Cardiology, Department of Internal Medicine National Taiwan University Hospital and National Taiwan University College of Medicine Taipei Taiwan Republic of China; ^6^ Division of Cardiology, Department of Medicine Taipei Veterans General Hospital Taipei Taiwan Republic of China; ^7^ Department of Internal Medicine, College of Medicine National Yang Ming Chiao Tung University Taipei Taiwan Republic of China; ^8^ Department of Cardiology Clinical Research Centre, Queen Elizabeth Hospital II Kota Kinabalu Malaysia; ^9^ Department of Cardiology Apollo Hospitals Chennai India

**Keywords:** amyloidosis, Asian patients, diagnosis, guidelines, healthcare resources, transthyretin amyloid cardiomyopathy

## Abstract

Transthyretin amyloid cardiomyopathy (ATTR‐CM) is a debilitating and life‐threatening condition with a heterogeneous clinical presentation. Recent guidelines from the United States and Europe have been published to guide clinical practice and to facilitate management conformity by covering current diagnostic and treatment strategies for patients with ATTR‐CM. These guidelines highlight the importance of an early diagnosis to optimize therapeutic outcomes, specifying the use of tests and imaging techniques to allow accurate, noninvasive diagnosis of ATTR‐CM. However, as regional practice variations across Asia may limit access to healthcare, availability of specific tests, and expertise in assessing diagnostic images, there is an ongoing need to provide an Asian perspective on these clinical guidelines. This review article provides practical recommendations for the diagnosis and monitoring of patients with ATTR‐CM in Asia, highlighting the need for additional guidelines to support a broad and diverse population, consider differing healthcare systems and diagnostic testing availability, and provide a flexible yet robust algorithm.

## 1 INTRODUCTION

Amyloidosis is a debilitating systemic disease characterized by the extracellular deposition of insoluble misfolded amyloid proteins in one or more organs.^1^, ^2^ Cardiac amyloidosis, an under‐recognized cause of heart failure (HF), leads to restrictive cardiomyopathy caused by extracellular deposition of proteins in the myocardium and is the major contributor to poor prognosis in patients with systemic amyloidosis.^2^, ^3^, ^4^ While more than 30 proteins can form the amyloid fibrils responsible for cardiac amyloidosis, monoclonal immunoglobulin light‐chain amyloid (AL) and transthyretin amyloid (ATTR) are the two main amyloid types that infiltrate the heart.^2^ Of note, ATTR can be found in patients with (hereditary type) or without (wild type) genetic defects.

While transthyretin amyloid cardiomyopathy (ATTR‐CM) has historically been considered rare, an estimation of “real‐life” prevalence remains a challenge because it is frequently under‐recognized by clinicians.^3^ In addition, ATTR‐CM leads to reduced quality of life and death.^5^ Following a diagnosis of ATTR‐CM in untreated patients, median survival remains poor.^6^, ^7^, ^8^ Guidelines from the American Heart Association (AHA) and European Society of Cardiology (ESC) Working Group on Myocardial and Pericardial Diseases have recently been published to guide clinical practice and to facilitate management conformity by covering current diagnostic and treatment strategies for patients with ATTR‐CM.^3^, ^9^ Of note, early identification of affected individuals has been highlighted as being a critical factor in the optimization of therapeutic outcomes.^3^ Guidelines from the Japanese Circulation Society Joint Working Group on the diagnosis and management of cardiac amyloidosis were published in 2020, with a simple staging system combining high‐sensitivity cardiac troponin T, brain natriuretic peptide (BNP), and estimated glomerular filtration rate, more recently reported to be useful in predicting prognosis in Japanese patients with wild‐type ATTR‐CM.^10^, ^11^ The Taiwan Society of Cardiology and the Society of Nuclear Medicine of the Republic of China support the application of technetium‐99m pyrophosphate ([99m]Tc‐PYP) scintigraphy in the diagnosis of ATTR‐CM.^12^ However, similar guidelines and recommendations are not available for other Asian countries.

Cardiac amyloidosis, including ATTR‐CM, is not widely recognized in Asian scientific literature suggesting that it may be underdiagnosed.^1^, ^13^, ^14^, ^15^, ^16^ A Japanese study has revealed an increasing trend in mortality associated with amyloidosis, and in particular a marked increase in mortality among men over the past 6 years.^17^ Moreover, while the AHA guidelines detail the use of readily available tests and imaging techniques to allow accurate, noninvasive diagnosis of ATTR‐CM (without confirmatory endomyocardial biopsies), regional practice variations across Asia may limit access to healthcare, availability of specific tests, and expertise in assessing diagnostic images. Consequently, the overall patient pathway may also vary between different healthcare systems.

This manuscript is primarily based on a discussion at an Expert Panel Meeting titled ‘Clinical recommendations to diagnose/monitor patients with ATTR‐CM in Asia', held virtually on March 26, 2021.

## 2 SUSPECTING ATTR‐CM

Given the nonspecific presenting symptoms of cardiac amyloidosis, diagnosis relies on a high index of clinical suspicion.^3^, ^9^, ^18^ “Red flags” refer to signs and symptoms of cardiac amyloidosis that support a high degree of suspicion, most of which can be identified from an initial physical examination and assessment of patient history, and which should prompt screening. A summary of possible red flags for cardiac amyloidosis is shown in Table 1. The presence of any of these in a patient should result in referral to specialized medical centers for further diagnostic workup by a cardiologist and/or neurologist.

**Table 1:** Extracardiac and cardiac red flags for cardiac amyloidosis

Extracardiac/cardiac	Red flag
Extracardiac	History of chronic diarrhea or constipation History of bilateral carpal tunnel syndrome Family history of systemic amyloidosis Signs of autonomic dysfunction (postural hypotension and diarrhea/constipation) Progressive unexplained weight loss Hand/feet numbness Enlarged tongue Signs of spinal stenosis especially in young adult
Cardiac	Signs/symptom of heart failure (dyspnea, fatigue, and edema) Family history of cardiomyopathy Recurrent postural dizziness, particularly after receiving low dose of anti‐hypertensive agents, e.g., beta‐blockers or angiotensin‐converting‐enzyme inhibitor/angiotensin II receptor blocker Signs of dysautonomia (orthostatic hypotension and bradycardia) Progressive “improvement” in blood pressure control in chronic hypertensive patients Unexplained cardiomegaly, combination of heart failure with neuropathy

Neurological red flags for ATTR‐CM include sensorimotor polyneuropathy (paresthesia and weakness), autonomic dysfunction (alternating diarrhea/constipation; orthostatic hypotension), and a family history of polyneuropathy, while orthopedic red flags include carpal tunnel syndrome, lumbar spinal stenosis, rupture of biceps tendon, and muscle weakness/sarcopenia. Table 2 summarizes differences in red flags between amyloidosis types which may help to support any suspicion of AL amyloidosis or ATTR amyloidosis (hereditary or wild‐type) in a patient.

**Table 2:** Symptoms of individuals presenting with systemic amyloidosis.^19^

	AL	Hereditary ATTR	Wild‐type ATTR
Thick LV, HFpEF	♦	♦	♦
Bilateral carpal tunnel	♦	♦	♦
Spinal stenosis	♦	♦	♦
Facial purpura, macroglossia	♦		
Atypical MGUS	♦		
Proteinuria, nondiabetic	♦		
ANS dysfunction, small fiber neuropathy	♦	♦	
Biceps rupture			♦

Abbreviations: AL, light‐chain amyloid; ANS, autonomic nervous system; ATTR, transthyretin amyloid; HFpEF, heart failure with preserved ejection fraction; LV, left ventricular; MGUS, monoclonal gammopathy of undetermined significance.

Available literature suggests that the main presenting pathologies of wild‐type ATTR are cardiomyopathy, carpal tunnel syndrome, and spinal canal stenosis, while patients with hereditary ATTR present with a higher incidence of gait instability, gastrointestinal symptoms, urinary incontinence, and neuropathic pain^14^, ^20^, ^21^; clinical experience suggests that these differences in presenting pathologies are also found in different populations of Asian patients.^22^, ^23^, ^24^, ^25^ Concomitant ATTR amyloidosis and severe aortic stenosis has been reported to be a relatively common finding in the elderly Indian population.^26^ For hereditary ATTR in the Asian population, Ala97Ser (p.A97S) appears to be the most commonly identified mutation^23^, ^24^; other identified mutations included ATTR‐V30M (p.Val50Met), ATTR‐R34T (p.Arg54Thr), ATTR‐S50I (p.Ser70Ile), ATTR‐H88R (p.His108Arg), and ATTR‐A97S (p.Ala117Ser).^23^ Of note, ATTR p.A97S has been described as cardiomyopathy as well as a polyneuropathic syndrome.^22^

A subsequent cardiac workup enables the identification of cardiac red flags, such as specific electrocardiography (ECG) features (low/relative low voltage vs. wall thickness) and echocardiography findings (aortic stenosis, HF with preserved ejection fraction [HFpEF], and relative apical sparing of longitudinal strain). Based on clinical experience, early red flag cardiac signs/symptoms for ATTR‐CM appear to be similar between patients in Asia and those in the USA and Europe, as detailed in the recent AHA guidelines and ESC Working Group Position Statement.^3^, ^9^

Panel recommendations for suspecting ATTR‐CM are shown in Table 3.

**Table 3:** Panel recommendations

Suspecting ATTR‐CM
Low voltage in ECG, specifically with respect to LV wall thickness, remains an important red flag for patients in Asia with ATTR‐CM (no available data to support a specific minimum value that should raise suspicion) ECG voltage can vary from low to normal, and even to high, in Asian patients.^27^, ^28^ However, clinical experience suggests that a “relative” low voltage (compared with LV wall thickness) is possibly the most important sign of cardiac amyloidosis Indices involving voltage strength/LV wall thickness/apical strain values may provide quantified measures which suggest the presence of amyloidosis (relative “apical sparing” in regional wall motion abnormality in strain image) While AF is common in cardiac amyloidosis, the presence of arrhythmia is too broad to be a designated red flag. However, AF and high degree atrioventricular block in patients with unexplained LVH may increase suspicion of cardiac amyloidosis^29^, ^30^ The presence of amyloid deposits identified during further testing in addition to LVH are suggestive of ATTR‐CM Aortic stenosis (particularly in those individuals with low‐flow low‐gradient aortic stenosis) in addition to RV thickening should also be considered as a red flag for ATTR‐CM Hypertension that resolves over time, and intolerance to angiotensin receptor blockers, angiotensin‐converting enzyme inhibitors, or beta‐blockers, are useful signs for suspecting ATTR‐CM^31^
Diagnosis of ATTR‐CM
While PYP scintigraphy is now quite widely available across most of Asia, scans may be positive even in AL amyloidosis^32^ or in patients with hypertrophic cardiomyopathy,^27^, ^33^ highlighting the need to test for serum and urine ALs concurrently with 99mTc‐PYP scintigraphy in patients with suspected ATTR‐CM AL amyloidosis and ATTR‐CM can co‐exist in the same patient, thus highlighting the need for parallel PYP scintigraphy and AL testing Scintigraphy and AL testing are dependent upon operator expertise and correct interpretation can be inconclusive and return false‐positive results (see *3.2. Potential barriers limiting choice of confirmatory diagnostic procedure in Asia*) Ambiguous results from PYP scintigraphy and AL testing may require an endomyocardial biopsy and further subtyping, such as mass spectrometry, to confirm a diagnosis of ATTR‐CM While abdominal fat biopsy or aspiration is cost‐effective and could be done before cardiac biopsy is considered, it lacks the sensitivity to exclude ATTR‐CM^34^ and other methods may be needed to differentiate clearly between ATTR‐CM and AL amyloidosis
Genetic testing of and screening for ATTR‐CM
As the prevalence of hereditary ATTR versus wild‐type ATTR appears to vary across Asia, genetic testing should always be performed, where available, unless the patient refuses. The panel recognizes that genetic testing is not readily available in some Asian countries/regions Potential screening of first‐degree family members (using genetic testing or at least a screening ECG) is recommended, although availability varies, with genetic counseling recommended, where available, following confirmation of hereditary ATTR

Abbreviations: AF, atrial fibrillation; AL, light‐chain amyloid; ATTR‐CM, transthyretin amyloid cardiomyopathy; ECG, electrocardiography; LV, left ventricular; LVH, left ventricular hypertrophy; PYP, pyrophosphate; RV, right ventricular.

The varying incidence of ATTR‐CM by gender across Asian countries suggests patient gender is not a suitable red flag. However, there have been reports of men having onset of ATTR at an earlier age compared with women.^15^ Ochi et al. recently reported that female patients with wild‐type ATTR had smaller left ventricular (LV) wall thicknesses, posterior wall thickness, and a higher LV ejection fraction compared with male patients^35^; however, the severity of HF did not differ between female and male patients. Age may be a more important indicator when assessing patients for possible ATTR‐CM. While data for Asian patients remain limited, hereditary ATTR has been reported in patients aged approximately 60 years of age in Taiwan,^15^, ^22^, ^36^ 50 years in South Korea,^37^ and clinical experience suggests ≥40 years in Japan. Limited data currently available on patient age in wild‐type ATTR‐CM suggest that age at diagnosis does not differ between female and male patients, although female patients tend to be older than male patients at the onset of cardiac symptoms.^35^ Clinical experience suggests that wild‐type ATTR‐CM is typically seen in older Asian patients aged ≥70 years.

## 3 DIAGNOSING ATTR‐CM

### 3.1 The diagnostic pathway for ATTR‐CM

**Figure 1:**
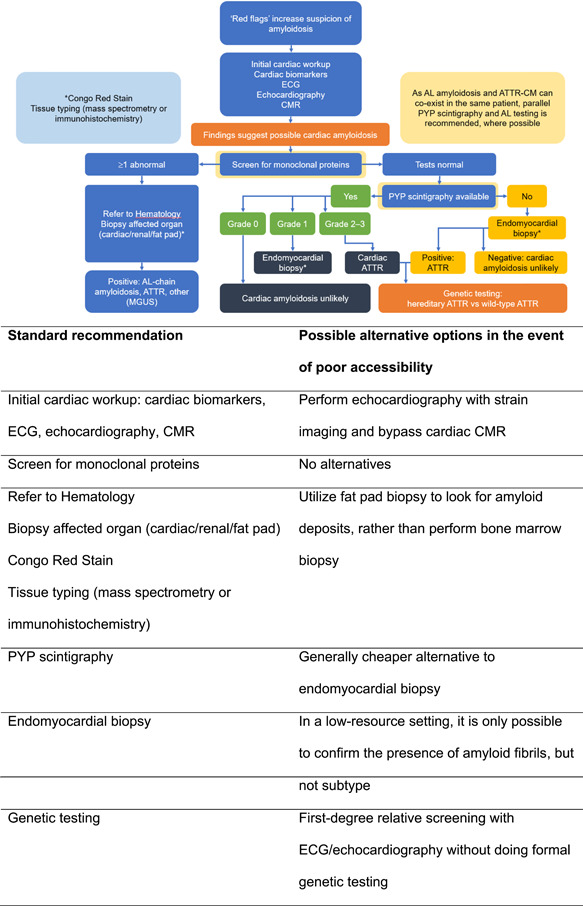
Diagnostic algorithm for ATTR‐CM in Asian patients: standard recommendations and alternative options. Adapted from Ash et al.^40^ AL, light‐chain amyloid; ATTR‐CM, transthyretin amyloid cardiomyopathy; CMR, cardiac magnetic resonance imaging; ECG, electrocardiography; MGUS, monoclonal gammopathy of undetermined significance; PYP, pyrophosphate

Initial steps in the diagnostic pathway for ATTR‐CM are the presence of red flags which suggest the presence of amyloidosis, followed by the initiation of the cardiac workup to assess the patient ECG and echocardiography (Table 4 and Figure 1). Echocardiography is widely available in Asia and is the initial imaging test of choice for diagnosis of cardiac amyloidosis. Infiltration of ventricular walls produces an appearance of hypertrophy with non‐dilated or small ventricles. Relative apical sparing of longitudinal strain is a key feature.^38^ The presence of any irregularities in myocardial contraction fraction and/or ejection fraction (caused by deformation of myocardium/shortening of myocardial fibers and measured by strain imaging) also warrants consideration. If results from initial echocardiography are unclear, the use of cardiac magnetic resonance imaging (CMR), where available, can provide a detailed assessment of the cardiac structure (including identification and quantification of LV hypertrophy [LVH]), function, and tissue characteristics. A real‐world analysis of CMR in a small number of patients demonstrated that transmural patterns of late gadolinium enhancement could distinguish ATTR from AL cardiac amyloidosis with a high level of accuracy.^41^ However, more recent publications suggest that classic imaging features on CMR, although typical for cardiac amyloidosis, are not specific enough to distinguish between AL and ATTR types.^42^, ^43^ Of note, CMR can be used as a screening tool for patients found to have unexplained LVH and should be employed in the setting of suspected hypertrophic or infiltrative cardiomyopathies to aid diagnosis.^44^ CMR has also played a role in differentiating between ATTR‐CM and the overlapping clinical phenotype of hypertrophic cardiomyopathy or infiltrative cardiomyopathy, given that these conditions can sometimes result in a positive [99m]Tc‐PYP scintigraphy scan.^27^, ^33^, ^44^ However, clinical experience suggests that the use of CMR versus PYP scintigraphy remains controversial given that while CMR is typically more widely available, PYP scintigraphy is less expensive and allows differentiation between amyloidosis types; however, CMR may have use as a “tie breaker” in cases of diagnostic ambiguity. Of note, given the importance of an early diagnosis in patients with ATTR‐CM and variable diagnostic testing availability, the use of the recently validated Kumamoto criteria comprising a scored combination of high‐sensitivity cardiac troponin T (≥0.0308 ng/ml), wide QRS interval (≥120 ms), and LV posterior wall thickness (≥13.6 mm), can raise the pre‐test probability for [99m]Tc‐PYP scintigraphy.^45^, ^46^ The importance of correctly differentiating between AL and ATTR amyloidosis should not be overlooked given that the AL type may have a more fulminant course and requires a different therapeutic approach to the ATTR type. There has also been a radical shift in how ATTR‐CM is being diagnosed following an initial cardiac workup which suggests the presence of cardiac amyloidosis.^3^ Previously, every patient had to undergo tissue (commonly endomyocardial) biopsy to confirm a diagnosis of ATTR‐CM. However, the current AHA diagnostic algorithm proposes the use of 99mTc‐PYP scintigraphy to replace invasive cardiac biopsy in diagnosing ATTR‐CM. Similarly, the ESC Position Statement proposes only noninvasive diagnostic approaches for ATTR‐CM via the initial use of 99mTc‐PYP, 99mTc‐3, 3‐diphosphono‐1,2‐propanodicarboxylic acid, or 99mTc‐hydroxymethylene diphosphonate scintigraphy.^9^

**Table 4:** Initial diagnostic approach for a patient in Asia suspected of having ATTR‐CM. Content‐based on clinical experience and available AHA/ESC guidelines.^3^, ^9^

Clinical approach	Key findings suggestive of ATTR‐CM
Red flags	Initial physical examination and patient history may support a suspicion of ATTR‐CM and lead to subsequent referral to a cardiac specialist
ECG	Specific ECG features that suggest ATTR‐CM include low/relative low voltage versus ventricular wall thickness
Echocardiography	Specific echocardiography findings that suggest ATTR‐CM: Appearance of hypertrophy with small/non‐dilated ventricles: a value of 12 mm is deemed to be the appropriate cutoff value to determine abnormal LV wall thickness in patients with ATTR‐CM* (although a value of ≥14 mm may be more specific in countries with limited resources) HFpEF Relative apical sparing of longitudinal strain (i.e., the ratio of apical longitudinal strain/average of mid and basal longitudinal strain >1.0), with high sensitivity (93%) and specificity (82%)^38^ Aortic stenosis (particularly in patients with low‐flow, low‐gradient severe aortic stenosis) Nonspecific, but characteristic findings: − Thickening of valves and interatrial septum − Sparkled appearance of the myocardium − Dilated atria − Pericardial effusion − Impaired diastolic function, with typically restrictive physiology in advanced stage

Abbreviations: ATTR‐CM, transthyretin amyloid cardiomyopathy; ECG, electrocardiography; HFpEF, heart failure with preserved ejection fraction; LV, left ventricular; LVH, left ventricular hypertrophy.

* LV wall thickening may be mild (<14 mm) or absent in the early stage of cardiac amyloidosis.^39^

Panel recommendations for the diagnosis of ATTR‐CM are shown in Table 3.

Figure 1 provides a proposed diagnostic algorithm for ATTR‐CM in Asian patients (based on AHA guidelines and the ESC Position Statement) which also considers possible alternative diagnostic pathways for where specific assessments or clinical expertise are not available (low‐ vs. high‐resource settings). For example, an initial cardiac workup in a high‐resource setting may include the assessment of cardiac biomarkers and ECG, along with the use of echocardiography and CMR, an alternative diagnostic approach in a low‐resource setting could include the use of echocardiography with stain imaging and bypass CMR.

### 3.2 Potential barriers limiting choice of confirmatory diagnostic procedure in Asia

It is important to recognize that each imaging modality used in the diagnosis and management of patients with infiltrative cardiomyopathies have both strengths and weaknesses, while also demanding a high level of awareness and expertise to allow the appropriate and correct interpretation of available diagnostic results.^47^ For example, classic imaging features on echocardiography and CMR, although typical for cardiac amyloidosis, are not specific enough to distinguish between AL amyloidosis and ATTR.^42^, ^43^ In addition, it is important to consider an individual hospital's capabilities and resources, while recognizing the wide disparities of diagnostic testing availability and clinical expertise both between and within Asian countries.

While endomyocardial biopsy may still be required to confirm the diagnosis of ATTR‐CM in some patients, this invasive procedure carries a certain amount of risk and should ideally be reserved for when the results from multimodality imaging are inconclusive, since it has a chance of producing a false negative result, along with the risk of bleeding complications. In addition to the routine Congo red stain, the use of endomyocardial biopsy also requires specific immunohistochemistry staining or mass spectrometry (the gold standard in amyloid protein subtyping) to differentiate between ATTR amyloidosis and AL amyloidosis, neither of which is widely available across Asia. Some centers may also lack the pathology expertise to correctly assess the biopsy sample and/or lack immunohistochemistry expertise to correctly identify ATTR amyloidosis. Endomyocardial biopsy may not be available in those centers which do not undertake cardiac transplantation or cardiothoracic surgery. Of note, most of the referral centers across Asia that do perform myocardial biopsies should have the capability to perform all relevant diagnostic imaging for ATTR‐CM.

Clinicians should be aware that all cardiac imaging techniques and biopsy procedures are operator dependent and require a high level of clinical expertise for accurate recognition of the features of ATTR‐CM. Varying clinical expertise in the selected diagnostic procedure may limit the number of robust diagnoses and potentially increase the number of false negatives from available results. For example, for scintigraphy, negative results may be reported even in patients with other highly suspicious clinical findings, such as echocardiography results. Thus, an equivocal result following scintigraphy in an otherwise clinically suspicious case may still require a biopsy to provide a more reliable finding. In addition, scintigraphy protocols can vary a lot between hospitals, highlighting the ongoing need to align practice within a country.^48^ Clinical experience also suggests expertise in interpreting imaging results continues to vary across Asia, which may result in misdiagnoses.

Genetic testing may not be available in some tertiary care centers in Asia, resulting in some patient blood samples being tested abroad and test‐result wait times of ≥1 month. For AL amyloidosis, clinical experience suggests that any delay in treatment will have prognostic significance and negatively affect patient outcome.

It is important to note that in some Asian countries, HF may be generally under‐managed or simply “not on the healthcare agenda,” with limited numbers of cardiologists in the public sector (compared with the private sector). Thus, there is an ongoing need to raise awareness of HF/ATTR‐CM and provide additional education for cardiologists across Asia, possibly via national HF groups/societies, where available. In addition, a positive diagnosis of ATTR‐CM may not necessarily lead to treatment in some Asian countries. For example, when patients in Thailand are diagnosed with ATTR‐CM, they do not receive recommended disease‐modifying therapies due to their current lack of availability, while tafamidis is available in India only on a humanitarian basis following regulatory compliance. As tafamidis is expensive where available, at the present time, some physicians may use other non‐standard/off label treatments that they have access to, such as diflunisal/doxycycline.

Parallel public and private healthcare systems might affect the availability of diagnostic testing and treatment programs in Asia. For example, profit‐driven private healthcare might be less motivated to develop an amyloidosis treatment program, or develop Tc‐PYP capabilities, as this is a relatively low‐volume investigation. As transplant centers are generally in the public system, private healthcare systems may have limited resources to perform cardiac biopsies. Clinicians should be aware of general similarities and differences between public and private parallel healthcare systems in their respective countries.

## 4 GENETIC TESTING AND SUBSEQUENT MONITORING OF ATTR DISEASE

According to the AHA guidelines and ESC Position Statement, if ATTR‐CM is identified, then genetic sequencing of the *TTR* gene is required to distinguish hereditary ATTR from wild‐type ATTR disease.^3^, ^9^

Panel recommendations for the genetic testing of and screening for ATTR‐CM are shown in Table 3.

Additional clinical evidence is needed to support a robust follow‐up strategy for those asymptomatic patients with genetically positive ATTR‐CM and their family members. Ueda et al. (2020) suggest that physical examination, biopsy, blood testing, renal function, blood pressure, ECG, echocardiography, and ophthalmologic assessment should be performed as a minimal level of examination annually, while in‐depth assessment of these parameters, along with PYP myocardial scintigraphy, should be performed every 3–5 years.^49^ In the absence of evidence and suitable biomarkers, clinical experience suggests that PYP screening or CMR, repeated at 3–5‐year intervals, or at the onset of symptoms, maybe a suitable approach. This monitoring strategy is similar to that used for those patients with confirmed ATTR‐CM and mild symptoms. The use of an annual ECG in these patients may also support the development of a clinical “snapshot” of the disease on an individual basis. While there are limited data to support the routine use of echocardiography or CMR to monitor asymptomatic patients with ATTR‐CM, echocardiography is widely available and affordable across Asia, and its use every 6 months–2 years may be appropriate in asymptomatic patients, or where there is a change in clinical status (e.g., functional decline); clinicians should specifically look for diastolic dysfunction, progressive LVH, and valvular dysfunction. The use of follow‐up echocardiography may be extended to 3–5 yearly intervals in resource‐limited clinical settings. The additional use of CMR every 3–5 years, where the patient and/or healthcare system can afford it, may provide a more detailed clinical snapshot of disease progression. There is currently little evidence to support initial monitoring at any specific age in asymptomatic family members, although first‐degree relatives of a patient diagnosed with ATTR‐CM should undergo screening, such as baseline imaging, at the earliest opportunity even in the absence of genetic testing. Of note, Ueda et al. suggest that predictive genetic testing for hereditary ATTR amyloidosis should be considered for adult (≥18 years) at‐risk relatives, given that this disorder has an adult‐onset, and no effective preventive therapies are currently available.^49^

Clinical experience in Asia suggests that initial baseline echocardiography should be undertaken in a family member around the time of the diagnosed index case (i.e., following confirmation of gene positivity). Baseline neurological and ophthalmological evaluations should also be undertaken. While there is no clinical evidence to support any specific follow‐up duration where the first screen is deemed to be normal, routine follow‐up echocardiography and eye examination every 3–5 years may be justified in adult patients or, alternatively, in younger family members, at the age of 10 years before the initial symptoms of the index case. In addition, additional neurological examination should be considered upon the development of any new symptoms.

It is important for clinicians to understand that the onset of cardiomyopathy may be earlier than HF in general.

## 5 ASSESSMENT OF TREATMENT RESPONSE AND DISEASE PROGRESSION

There remains no accepted definition of ATTR‐CM progression or response to therapy.^3^ Treatment response can only really be assessed by frequency of hospitalization for HF, frequency of episodes of HF, diuretic use, 6‐minute walking distance, and, potentially, improvement in cardiopulmonary maximal oxygen consumption. Garcia‐Pavel et al. suggest that the minimum requirement for assessing ATTR‐CM progression should comprise one marker from the domains of (i) clinical and functional endpoints (e.g., increase in HF‐related hospitalization), (ii) biomarkers and laboratory markers (e.g., 30% increase in troponin), and (iii) imaging and electrocardiographic parameters (e.g., new‐onset conduction disturbance).^50^ In addition, assessment of cardiac disease status should form part of a multiparametric evaluation that considers progression, stability, or improvement of other involved systems in ATTR amyloidosis.

Clinical experience in Asia suggests that a 6‐minute walk test is a useful tool for monitoring treatment response and progression in patients without significant concomitant neuropathy, given that it is cheap, reproducible, and universally available. Concentrations of cardiac biomarkers, such as serum N‐terminal pro‐BNP and serum cardiac high‐sensitivity troponin, are also useful monitoring tools. The role of imaging modalities in evaluating response to therapy has not yet been established. However, ongoing studies are currently assessing the value of CMR in monitoring patients with ATTR‐CM.^2^, ^51^ Echocardiography may also be used to monitor disease (every 6 months). Plasma TTR concentration and pre‐albumin levels are also potentially valuable markers, although the use and availability of plasma TTR concentration appears to vary both by center and country and is not widely available across Asia. Extracellular volume assessment by CMR is an emerging technique for monitoring treatment response and progression, and is independently predictive of both prognosis and disease progression^52^, ^53^; this approach provides early detection and evaluation of amyloid infiltration in the myocardium. Evidence to demonstrate the role of cardiopulmonary exercise stress testing in monitoring patients with ATTR‐CM remains scarce and, moreover, may not be technically practical for those patients living with this chronic deteriorating disease.

The frequency of monitoring for response to treatment and disease progression in Asia should reflect any practical considerations for patients with ATTR‐CM, such as ease of access to the medical center. Clinical monitoring should ideally be scheduled (at a tertiary center/center with amyloid expertise) every 3–4 months in patients with symptomatic, stable disease, while this should increase in frequency to every 1–2 months in those with progressive disease given the increased possibility of rapid physical deterioration in these individuals; where medical facilities may be limited or patients reside in remote locations, monitoring should still be undertaken at least every 6 months as a minimum requirement. In addition, as the vast majority of patients ATTR‐CM will have concomitant neurological involvement, any limit(s) in daily activity should be carefully considered when determining the frequency and type of disease monitoring.

As ATTR‐CM progresses and the patient becomes more debilitated with limited longevity, clinicians may wish to stop performing advanced imaging and simply let the local medical facility manage patient symptoms. Advanced care plans/palliative care should be discussed with patients given the prognosis (3–5‐year life expectancy following diagnosis) of ATTR‐CM.

## 6 CONCLUSIONS

In Asia, educating patients, general clinicians, and cardiologists about ATTR‐CM remains important to increase understanding of this under‐diagnosed condition. Of note, there should be increased emphasis on developing an ‘amyloidosis‐aware mindset’ in physicians, particularly echocardiographers.

While the timely diagnosis of cardiac amyloidosis is challenging, a high level of clinical suspicion using red flags and genetic testing, along with suitable additional testing options (dependent upon availability) are key for the early diagnosis of ATTR‐CM. The emergence and increasing availability of newer noninvasive imaging techniques can be used to provide prognostic information, potentially obviating the need for endomyocardial biopsy in some patients.

The diagnostic pathway for patients in Asia with suspected ATTR‐CM needs to acknowledge differences in country‐specific/regional healthcare systems, along with the availability of subsequent testing and sufficient operator expertise required to confirm a diagnosis. Of note, clinicians in Asia need to be mindful of equivocal and ‘imperfect' scenarios in the ATTR‐CM diagnostic journey, where testing/imaging results are ambiguous or may be confused by the presence of complex comorbidities. Importantly, there is an ongoing need for multi‐institutional/country collaborations to optimize the diagnosis of ATTR‐CM in a region such as Asia. While a patient registry for ATTR‐CM has been created in Singapore,^54^ there is an ongoing need for other Asian countries to do the same to allow patient data to be shared between them and support robust research into ATTR‐CM based on a large, Asian patient population. In the future, a possible collaboration between established registries (such as the Transthyretin Amyloidosis Outcomes Survey registry) and Asian registries could allow the comparison of patient characteristics and diagnostic pathways/outcomes, with the aim of sharing knowledge to optimize the diagnosis and management of patients with ATTR‐CM.

In summary, guidelines for the diagnosis and management of ATTR‐CM in Asia should support a broad and diverse population, consider differing healthcare systems and diagnostic testing availability, and provide a flexible yet robust algorithm.

## AUTHOR CONTRIBUTIONS

All authors take responsibility for all aspects of the reliability and freedom from bias of the recommendations presented and their discussed interpretation. All authors have made substantial contributions to all of the following: (1) assessment and interpretation of available literature, with subsequent recommendations, (2) drafting the article or revising it critically for important intellectual content, and (3) final approval of the version to be submitted.

## CONFLICTS OF INTEREST

Weiqin Lin has received advisory board fees/honoraria and research funding from Pfizer. Pairoj Chattranukulchai has no potential conflicts of interest to disclose. Yen‐Hung Lin has received research grants from Pfizer and speaker honoraria from Pfizer, MSD, Bayer, Novartis, Daiichi Sankyo, Taiwan Tanabe, Sanofi, Johnson & Johnson, Boehringer Ingelheim, AstraZeneca, Boston Scientific, Abbott, and Medtronic. Wen‐Chung Yu has acted as a consultant for Pfizer and Alnylam. Abraham Oomman has no potential conflicts of interest to disclose. Houng‐Bang Liew has no potential conflicts of interest to disclose. Alex PW Lee has received research grants and speaker honoraria from Pfizer.

## Data Availability

Data sharing in not applicable to this article as no new data were created or analysed in this study.
